# Plasma exosomal miRNA expression and gut microbiota dysbiosis are associated with cognitive impairment in Alzheimer’s disease

**DOI:** 10.3389/fnins.2025.1545690

**Published:** 2025-02-19

**Authors:** Kaihao Lin, Wenxia Lin, Zhikai Guo, Cuihong Chen, Liang Chen, Xianbin Cai

**Affiliations:** ^1^Department of Gastroenterology, The First Affiliated Hospital of Shantou University Medical College, Shantou, China; ^2^Neurology Department, The First Affiliated Hospital of Shantou University Medical College, Shantou, China; ^3^Department of Computer Science and Technology, College of Mathematics and Computer, Shantou University, Shantou, China

**Keywords:** Alzheimer’s disease, cognitive impairment, exosomal miRNA, gut microbiota, metagenomic sequencing

## Abstract

**Introduction:**

The gut microbiota composition and the expression profiles of microRNAs (miRNAs) in the brain tissue, cerebrospinal fluid, and blood of patients with Alzheimer’s disease (AD) differ significantly from those with normal cognition function. The study aimed to initially explore the relationship between plasma exosomal microRNAs, gut microbiota, and cognitive impairment, providing insights into the pathogenesis and treatment of AD.

**Methods:**

The study enrolled 8 participants with AD and 8 participants with normal cognition. The Mini-Mental State Examination (MMSE) was utilized to evaluate cognitive function. High-throughput sequencing was used to identify differentially expressed miRNAs in plasma exosomes, while metagenomic sequencing was employed to detect differences in the abundance of gut microbiota. Furthermore, the associations among them were analyzed.

**Results:**

Four exosomal miRNAs and 14 microbiota taxa, which exhibited differential expression and abundance, respectively, in comparison between AD group and normal cognition group, were identified to be significantly associated with MMSE scores. Notably, the abundance of potential probiotics, including *Faecalibacterium prausnitzii*, *Roseburia intestinalis* and *Roseburia inulinivorans*, which was decreased in AD patients, exhibited positive correlations with specific exosomal miRNAs: *Roseburia intestinalis* correlated with miR-3120-3p and miR-6529-5p; *Roseburia inulinivorans* correlated with miR-3120-3p, miR-6529-5p and miR-124-3p; *Faecalibacterium prausnitzii* correlated with miR-3120-3p.

**Discussion:**

The study revealed a close association among gut microbiota, plasma exosomal miRNAs, and cognitive impairment in AD, and suggested that specific components of gut microbiota and exosomal miRNAs may serve as potential biomarkers and therapeutic targets for AD on the microbiota-gut-brain axis.

## 1 Introduction

Alzheimer’s disease (AD) is the primary cause of dementia. As the global population ages, the prevalence of AD is on the rise, imposing a considerable strain on both families and society at large (Bacigalupo et al., 2018). AD is a neurodegenerative disease characterized primarily by neuritic plaques, neurofibrillary tangles, and the degeneration and death of neurons (McKhann et al., 2011; Jack et al., 2018). However, the precise etiology of AD remains ambiguous. A study by Montagne et al. utilized advanced imaging techniques to reveal that the breakdown of blood–brain barrier (BBB) in the hippocampus was an early event in aging humans and was more pronounced in individuals with cognitive impairment, suggesting that disruption of the BBB may contribute to early stages of AD (Montagne et al., 2015).

Exosomes can easily cross the BBB due to their small size (typically between 30 and 150 nm in diameter) and cell-like membrane structure (Wood et al., 2011). Neural cells produce and release exosomes, which can traverse the BBB and thus be detected in both blood and peripheral fluids (Kanninen et al., 2016). Exosomes can carry and protect microRNAs (miRNAs) from degradation. miRNAs are short, non-coding RNAs, about 20 to 25 nucleotides in length, that regulate gene expression by binding to specific mRNAs (Inui et al., 2010). Up to 70% of miRNAs are expressed in the human nervous system, and abnormal miRNA expression is associated with the pathogenesis of AD (Nowak and Michlewski, 2013). These miRNAs participate in the pathogenesis of AD by regulating and influencing key molecules such as microtubule-associated protein tau, amyloid precursor protein (APP), and *β*-site APP-cleaving enzyme 1 (BACE1) (Wang et al., 2023). Exosomal miRNA expression in plasma was found to change in patients with early cognitive impairment and AD, and some exosomal miRNAs were considered as potential biomarkers for AD (Yang et al., 2018; Jia et al., 2022; Duan et al., 2024). For example, miRNAs such as miRNA-135a and miRNA-384 can inhibit the production of amyloid-beta (Aβ) (Liu et al., 2014c, 2014b), while miRNA-193b can downregulate the expression of APP (Liu et al., 2014a). Therefore, changes in the expression profile of plasma exosomal miRNAs can reflect pathological changes in the central nervous system, serving as biomarkers for early diagnosis and disease progression monitoring of AD (Wang et al., 2023).

The intestine and brain are closely linked through the microbiota-gut-brain axis. Dysregulation of the gut microbiota can contribute to the pathogenesis and progression of AD by exacerbating immune senescence, oxidative stress, cytokine secretion, and neuroinflammation (Leblhuber et al., 2020). In AD patients, the diversity of gut microbiota shows a downward trend, and this change comes at the expense of anti-inflammatory probiotics, while leading to a significant increase in pro-inflammatory microflora (Varesi et al., 2022). The study by Vogt et al. (2017) reported that the abundances of *Firmicutes* and *Bifidobacterium* were significantly decreased in the feces of AD patients, whereas the abundance of *Bacteroidetes* was markedly increased. Lipopolysaccharide, primarily derived from *Bacteroides* and *Prevotella*, can cross the gut barrier and migrate to microglia, activating the NF-κB signaling pathway and regulating the expression of pro-inflammatory miRNAs (such as miR-146a and miR-155), thereby playing a crucial role in the pathogenesis of AD (Hill et al., 2015; Kim et al., 2021). Additionally, amyloids derived from gut microbiota may accumulate in the brain by translocating from the intestine, which may lead to an increase in the pro-inflammatory miRNA-34a levels and a suppression of phagocytosis mediated by TREM2, thus promoting the accumulation of Aβ42 peptides (Zhao and Lukiw, 2013; Zhao et al., 2015). It has been reported that exosomes originating from the gut microbiota of AD patients can cause tau protein to become overly phosphorylated and aggregated *in vitro*, suggesting a possible mechanism of disease progression (Haas-Neill and Forsythe, 2020). Furthermore, aging is associated with increased vulnerability of the human gastrointestinal tract and BBB, which may enable the translocation of microbiota-derived neurotoxins from the gut into the bloodstream (Tran and Greenwood-Van Meerveld, 2013; Man et al., 2014; Montagne et al., 2015; Qi et al., 2017). This, in turn, can provoke systemic inflammation, thereby exacerbating the destruction of the BBB and accelerating neurodegenerative changes in the nervous system. Recent research has shown that participants with mild cognitive impairment exhibited higher abundances of *Proteobacteria* and *Gammaproteobacteria*, and these abundances were correlated with serum levels of let-7g-5p, miR-107, and miR-186-3p (Zhang X. et al., 2021). The study highlighted the potential of gut microbiota combined with serum miRNAs as biomarkers for cognitive impairment.

The crosstalk between gut microbiota and miRNAs is closely related to AD. Dysregulation of the gut microbiota can mediate changes in miRNA expression in brain tissue, thereby influencing the pathogenesis and progression of AD (Ayyanar and Vijayan, 2024). However, few studies have explored the correlation between alterations in gut microbiota and changes in plasma exosomal miRNA expression in AD patients. Therefore, this study aimed to preliminarily explore the association between them. Our research findings revealed a decreased abundance of potential probiotics in AD patients, including *Faecalibacterium prausnitzii*, *Roseburia intestinalis* and *Roseburia inulinivorans*, which positively correlate with certain exosomal miRNAs (such as miR-3120-3p, miR-6529-5p, or miR-124-3p). This suggested that specific components of both the gut microbiota and exosomal miRNAs could be potential biomarkers and therapeutic targets for AD on the microbiota-gut-brain axis.

## 2 Materials and methods

### 2.1 Participant recruitment

The participants enrolled in this study were volunteers who received medical treatment or health examination in the First Affiliated Hospital of Shantou University Medical College from January 2021 to October 2022. All patients in the AD group met the clinical diagnosis of AD according to the 1984 NINCDS-ADRDA criteria (McKhann et al., 1984). To exclude cognitive impairments caused by other etiologies as much as possible and minimize the interference of other factors on the research results, the exclusion criteria for AD group included: (1) conditions such as altered consciousness, fatal diseases, drug poisoning, etc.; (2) a history of gastrointestinal tumors or inflammatory bowel diseases; (3) malignant anemia; (4) thyroid diseases that may affect cognition (Tan et al., 2008); (5) psychiatric disorders such as depression, that may lead to secondary dementia; (6) other brain diseases, including central nervous system infections, recent or old cerebrovascular accidents, hydrocephalus, subdural hematomas, Parkinson’s disease and Parkinson’s syndrome, Huntington’s disease, Creutzfeldt-Jakob disease, brain tumors causing dementia, or severe cerebral white matter rarefaction; (7) long-term exposure to heavy metals or chemicals in work and (or) living environments. The cognitively normal health control group met the following criteria: (1) possessing normal daily life cognitive abilities with no cognitive impairments; (2) having no relevant family history of inherited diseases affecting cognition, or a family history of AD in first-degree relatives; (3) having no blood relation to any member of the AD group.

### 2.2 Clinical data collection

The fundamental clinical data of the enrolled participants were collected, including age, gender, educational background, fasting blood glucose levels, indicators of liver and kidney function, as well as blood lipid profiles. Cognitive abilities were assessed using the Chinese adaptation of the Mini-Mental State Examination (MMSE) (Folstein et al., 1975; Katzman et al., 1988), which evaluates orientation, memory, attention, calculation, recall, and language, among other domains, with a total score of 30 points. The lower the score, the more severe the cognitive impairment. Participants in this study were required to meet the following clinical criteria: (1) no use of probiotics, antibiotics, or proton pump inhibitors in the past month; (2) no history of alcohol, drug, or substance abuse; (3) no significant digestive system symptoms such as jaundice, poor appetite, or hepatosplenomegaly; (4) no current acute gastroenteritis, intestinal tuberculosis, or other intestinal infections; (5) no severe conditions affecting gastrointestinal metabolisms, such as gastrointestinal bleeding, hepatitis, or liver damage.

### 2.3 Extraction and identification of plasma exosomes

**Figure 1:**
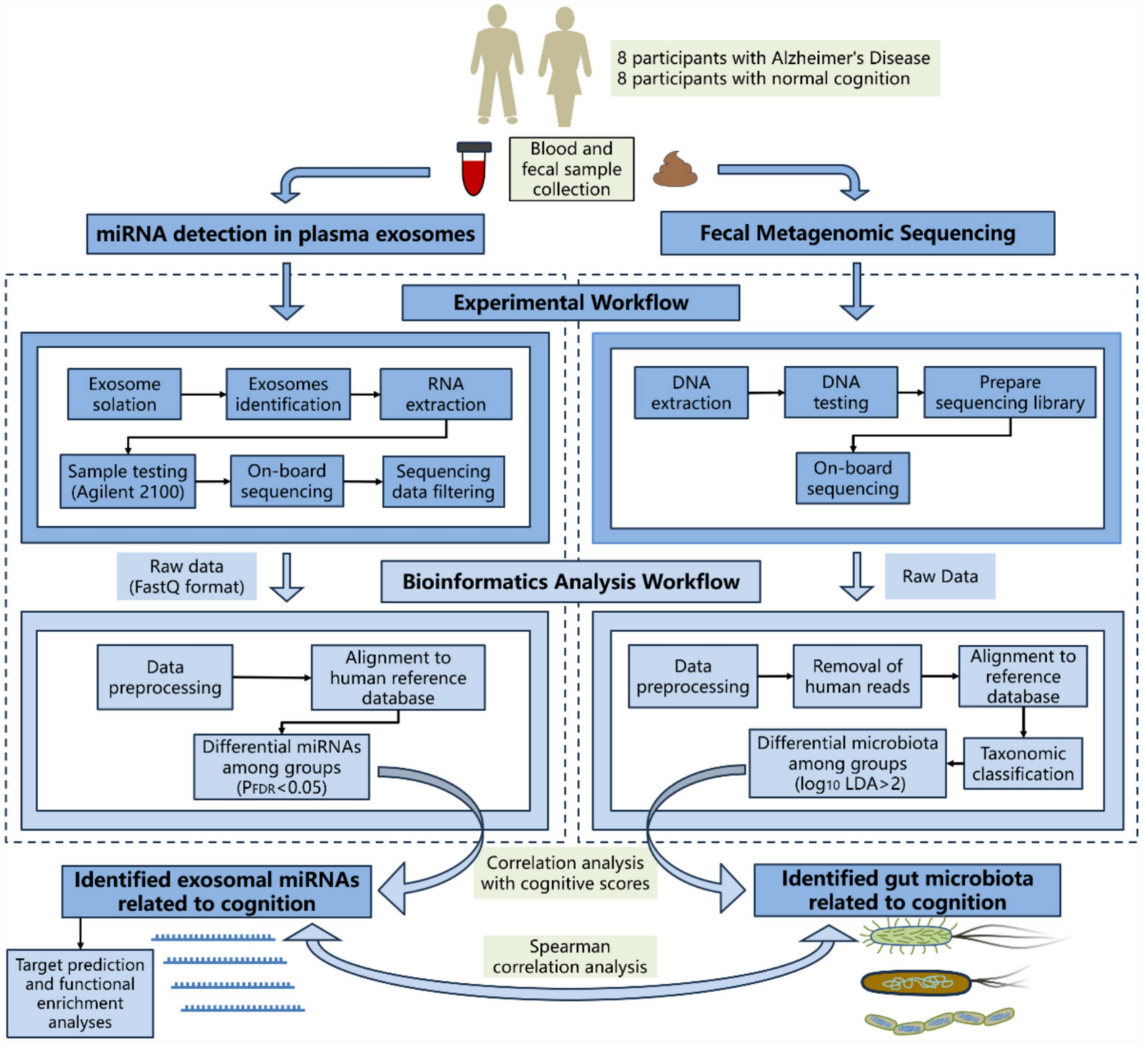
Brief flowchart of sequencing and analysis.

**Figure 2:**
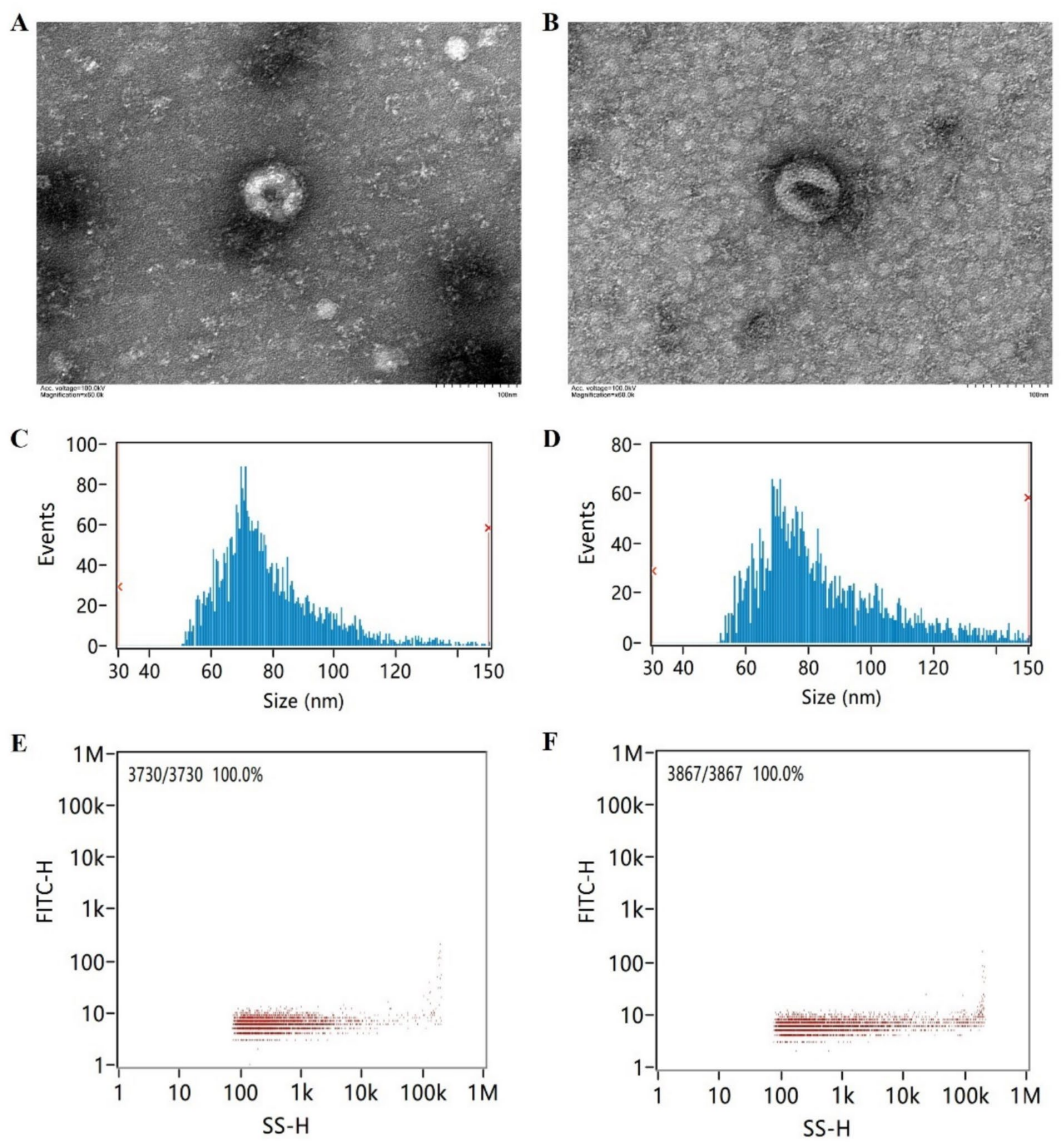
Electron microscopy imaging, size distribution, and concentration of exosomes. **(A)** Exosome electron microscopy image of the AD patient. **(B)** Exosome electron microscopy image of the cognitively normal participant. **(C)** Exosome size distribution of the AD patient. **(D)** Exosome size distribution of the cognitively normal participant. **(E)** Exosome concentration schematic of the AD patient. **(F)** Exosome concentration schematic of the cognitively normal participant.

The brief flowchart for the experimental operation and data analysis process of the whole study is shown in Figure 1. Fasting peripheral blood was drawn from participants using EDTA-coated tubes in the morning. The samples were centrifuged at 3,000 × *g* for 15 min at 4°C, then the supernatant was collected and stored at −80°C. After incubating the plasma samples at 37°C or room temperature until they were completely thawed, they were centrifuged at 10,000 × *g* for 15 min at 4°C. The supernatant was extracted and treated with Proteinase K (Life Technologies). Subsequently, incubated with total exosome isolation from plasma and centrifuged at 10,000 × *g* at 4°C to precipitate. The supernatant was then discarded, and the exosome-containing precipitate at the bottom of the tube was resuspended in PBS. Randomly select one case from both the AD group and the control group for exosome identification. Diluted exosome suspensions were deposited onto copper grids for precipitation. After treatment with phosphotungstic acid, transmission electron microscope images of the exosomes were obtained using the electron microscope (HITACHI, HT-7800) (Figures 2A,B). The size and concentration of the exosomes were measured using the Nanoparticle Size Analyzer (Flow NanoAnalyzer) (Figures 2C–F). The blots were incubated overnight with antibodies against CD63, CD81, TSG101, and Calnexin, all of which were diluted to 1:1000 and sourced from Abcam. After washing with TBST, secondary antibodies (diluted 1:5000) were added for 1 h. Finally, protein detection was performed using chemiluminescence (Bio-Rad ChemiDoc) (Supplementary Figure S1).

### 2.4 Isolation and sequencing of exosomal RNA

RNA was extracted from the exosome suspension through a series of steps, including QIAzol lysis, chloroform/isopropanol (24:1) extraction, ethanol precipitation, and column purification, ultimately obtaining the RNA elution product. The integrity and concentration of the samples were assessed using an Agilent 2,100 bioanalyzer, while the level of salt ion contamination was quantified with a NanoDrop spectrophotometer. The exosomal miRNA was sequenced using the DNBSEQ platform. After removing adapters, low-quality tags, and fragments shorter than 18 nucleotides, the raw sequencing data were acquired in FastQ format. The isolation and identification of exosomes, as well as the separation and sequencing of RNA, were carried out by Shenzhen BGI Genomics Co., Ltd.

### 2.5 Analysis of plasma exosomal miRNA sequencing data

The plasma exosomal miRNA sequencing data was initially subjected to a reliability evaluation of the reads by FastQC software (version 0.11.9) for quality assessment. Adapter sequences were thoroughly assessed for purity, and the dataset was refined using Trimmomatic (version 0.39) to remove low-quality sequences, resulting in a clean dataset. Subsequently, quality control was conducted once again utilizing FastQC. The clean data was aligned to human microRNAs (version 22) from the miRBase database^1^ and the GRCh38 human genome using Bowtie (version 1.3.1) and miRDeep2 software (version 0.3.1) to calculate the absolute number of reads aligned to human microRNAs.

The DESeq2 package (version 1.34.0) of R software was used to perform differential expression analysis of microRNAs. The DGEobj.utils package of R was utilized to obtain expression levels in reads per million (RPM) format based on the reads counts of plasma exosomal microRNAs. The gender (male/female) and age groups (50–65, 65–75, and ≥75 years) of participants were considered as batch variables to compare the expression levels of miRNAs between two groups. Using the Benjamini-Hochberg method to control the False Discovery Rate (FDR) (Benjamini and Hochberg, 1995), a threshold of <0.05 was used to determine statistical significance for identifying differentially expressed miRNAs in large-scale analyses. The search and prediction of target genes for plasma exosomal microRNAs were performed utilizing miRWalk (version 3)^2^. The identified target genes underwent Gene Set Enrichment Analysis (GSEA) and further functional enrichment pathway analysis using KEGG, Reactome, and Gene Ontology (GO). Functional pathways with an adjusted *p*-value less than 0.05 were selected as significantly enriched results.

### 2.6 Analysis of gut microbiota

Fecal samples were collected following a strict procedure. Samples were added to a preservative solution, thoroughly mixed, and stored in an ultra-low temperature freezer at −80°C. The extraction of DNA from microbial cells within fecal samples was performed utilizing the CTAB protocol. The concentration, purity, and integrity of the extracted DNA were then assessed using either Qubit 2.0 or Agilent 5,400. Qualified DNA samples were utilized to construct libraries with the NEB Next^®^Ultra™ DNA Library Prep Kit for Illumina (United States). Upon achieving library qualification, the indexed samples were subjected to clustering using the cBot Cluster Generation System, with the Illumina PE Cluster Kit (United States). Metagenomic sequencing of the samples was conducted using the Illumina Novaseq platform, resulting in raw metagenomic data. Subsequently, the raw data was subjected to preprocessing with Kneaddata software, which included adapter sequences, low-quality sequences (with a default quality score threshold ≤20), and sequences with a final length of less than 50 base pairs. The Bowtie2 software (version 2.35.5.1) was employed to eliminate host genomic sequences, while FastQC was utilized for quality assessment. The Kraken2 tool was utilized to align the sequence numbers of species present in the samples, and subsequently, Bracken was employed to forecast their relative abundances. The HUMAnN2 software was employed to align sequences that had undergone quality control and host removal with the protein database (UniRef90), utilizing DIAMOND to obtain annotation data from various functional databases and a relative abundance table for species (bacterial phyla) across different taxonomic levels. The LEfSe analysis of the fecal metagenomics data was conducted at the family, genus, and species levels, employing Hutlab’s Galaxy platform^3^. The Linear Discriminant Analysis (LDA) scores for various bacteria were calculated, and bacteria with an absolute value of the logarithm (base 10) of their LDA scores exceeding 2 were recognized as significantly distinct between groups at each taxonomic level (Segata et al., 2011). The above steps were primarily completed by the laboratory of Shenzhen Weikengmeng Technology Group Co., Ltd.

### 2.7 Statistical analysis

Given the limited sample size, the baseline data of the participants were initially evaluated for normality using the Shapiro–Wilk test by SPSS (version 25). For normally distributed data, the results were reported using mean ± standard deviation format, and statistical comparisons between groups were conducted using independent *t*-tests. For non-normally distributed data, the median (along with the 25th percentile and 75th percentile) was used, and the Wilcoxon rank-sum test was conducted to compare differences between groups. In correlation analysis, considering the small sample size of this study, the software SPSS 25.0 was used to test for normality using the Shapiro–Wilk test. If the variables exhibited a normal distribution, Pearson correlation analysis was applied. If not, Spearman correlation analysis was utilized. The correlation coefficient was considered statistically significant if the *p*-value was less than 0.05.

## 3 Results

### 3.1 Clinical data

A total of 11 individuals were planned to be included in the AD group and 9 in the healthy control group. However, 3 volunteers from the AD group did not complete the head magnetic resonance imaging (MRI) examination, and 1 volunteer from the healthy control group, who underwent a head MRI, was found to have a subacute pontine infarct. Finally, the study included 8 participants in the AD group and 8 in the healthy control group, maintaining a gender ratio of 1 male to 3 females. Apart from differences in cognitive scale scores (MMSE scores) and blood albumin levels, no statistically significant differences were found in the baseline characteristics of the two groups (Table 1).

**Table 1:** Baseline characteristics of the AD group and control group.

	AD group	Control group
Age (year)	71.3 ± 8.4	64.4 ± 8.3
Educational attainment (year)	4.5 ± 4.0	6.0 ± 4.5
Fasting blood glucose (mmol/L)	6.21 ± 1.58	6.10 ± 0.48
Direct bilirubin (μmol/L)	2.25 ± 0.48	2.13 ± 0.89
Indirect bilirubin (μmol/L)	9.79 ± 3.91	10.02 ± 3.35
Total bilirubin (μmol/L)	12.04 ± 4.00	12.15 ± 4.18
Triglycerides (mmol/L)	1.19 (0.75, 1.45)	1.21 (0.96, 1.42)
Total cholesterol (mmol/L)	5.85 ± 1.43	5.94 ± 1.35
High-density lipoprotein cholesterol (mmol/L)	1.35 ± 0.41	1.46 ± 0.32
Low-density lipoprotein cholesterol (mmol/L)	3.61 ± 1.13	3.45 ± 0.91
Aspartate aminotransferase (U/L)	24.11 ± 6.11	24.85 ± 5.62
Alanine aminotransferase (U/L)	18.35 ± 3.74	18.28 ± 6.33
Gamma-glutamyltransferase (U/L)	17.98 (13.61, 25.43)	25.06 (18.71, 27.53)
Lactate dehydrogenase (U/L)	210.00 (162.75, 227.25)	210.00 (182.50, 227.25)
Cholinesterase (kU/L)	7.72 (6.71, 8.92)	8.03 (7.30, 10.60)
Albumin (g/L)*	40.44 ± 2.67	43.36 ± 2.26
Globulin (g/L)	32.43 ± 3.55	30.13 ± 3.55
Total protein (g/L)	72.87 ± 2.90	73.48 ± 2.51
Albumin/Globulin ratio	1.27 ± 0.20	1.46 ± 0.25
Serum creatinine (μmol/L)	69.72 (65.73, 82.52)	74.97 (66.16, 74.97)
Uric acid (μmol/L)	348.53 ± 117.60	400.64 ± 81.37
MMSE score (score)*	14.5 (4.5, 14.5)	28.88 (27, 30)

The variables in the two groups that both satisfied the normal distribution were presented as mean ± standard deviation; otherwise, they were presented by median (25th percentile, 75th percentile). *P < 0.05 (*t*-test for normally distributed data, Wilcoxon rank-sum test for non-normal distributions).

### 3.2 Correlation between plasma exosomal miRNA expression and cognitive function

**Figure 3:**
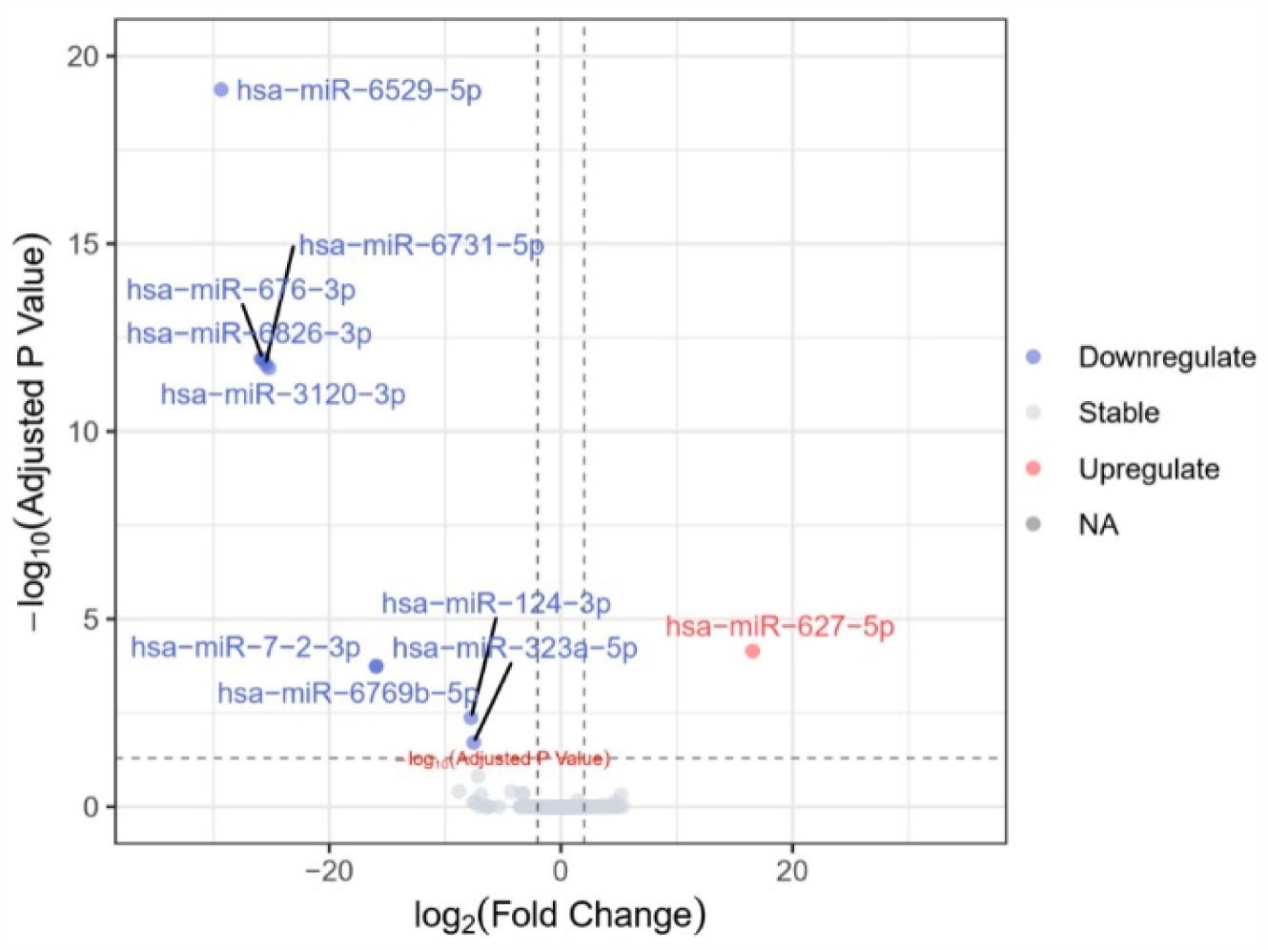
Volcano plot of differential expression of plasma exosomal microRNAs.

A total of 10 miRNAs with differential expression between groups were identified (*P*_FDR_ < 0.05) (Table 2 and Figure 3). Among them, only hsa-miR-627-5p was upregulated in AD patients, while the remaining 9 miRNAs were downregulated in AD patients. Spearman correlation analysis revealed that only hsa-miR-6529-5p (*r* = 0.580, *p* < 0.05), hsa-miR-3120-3p (*r* = 0.580, *p* < 0.05), hsa-miR-124-3p (*r* = 0.562, *p* < 0.05), and hsa-miR-323a-5p (*r* = 0.507, *p* < 0.05) were significantly correlated with MMSE scores (Table 2).

**Table 2:** Exosomal microRNAs with significant differences between case group and control group.

microRNA	log_2_FoldChange	*P*-value	*P*_FDR_	Expression in AD patients	Correlation coefficient with MMSE score^†^
hsa-miR-6529-5p	−29.33	1.06E-22	7.82E-20	Down	0.580*
hsa-miR-6826-3p	−25.89	3.27E-15	1.16E-12	Down	0.458
hsa-miR-676-3p	−25.75	4.71E-15	1.16E-12	Down	0.458
hsa-miR-6731-5p	−25.49	8.71E-15	1.61E-12	Down	0.458
hsa-miR-3120-3p	−25.2	1.39E-14	2.05E-12	Down	0.580*
hsa-miR-627-5p	16.56	5.76E-07	7.10E-05	Up	−0.029
hsa-miR-6769b-5p	−15.94	1.95E-06	1.80E-04	Down	0.314
hsa-miR-7-2-3p	−15.94	1.95E-06	1.80E-04	Down	0.314
hsa-miR-124-3p	−7.75	5.17E-05	0.004	Down	0.562*
hsa-miR-323a-5p	−7.53	2.63E-04	0.019	Down	0.507*

^†^Spearman correlation analysis; *P-value < 0.05.

### 3.3 Prediction of miRNA target genes and their functions

The target genes of the differentially expressed miRNAs associated with MMSE scores were predicted, and functional enrichment analysis was subsequently performed (Supplementary Table S1). KEGG analysis revealed the association of the predicted target genes of miR-3120-3p with neurotrophin signaling pathway and bacterial invasion of epithelial cells. GO analysis showed enrichment of target genes in various functional categories, including protein phosphorylation, nervous system development, protein K48-linked ubiquitination, c-Jun N-terminal kinase (JNK) cascade, activation of JUN kinase activity, among others. The predicted target genes of hsa-miR-124-3p may play crucial roles in signal transduction, transcriptional regulation, protein modification, and localization. The predicted target genes of hsa-miR-6529-5p may play significant roles in multiple aspects, including the development and function of the nervous system, cellular signal transduction, protein modification, and cell proliferation. Additionally, the enrichment results of the predicted target genes of miR-323a-5p suggested that they are associated with the APP catabolic process. Furthermore, the predicted target genes of these four miRNAs were all enriched in the soluble *N*-ethylmaleimide-sensitive fusion factor attachment protein receptor (SNARE) complex.

### 3.4 LEfSe analysis of gut microbiome

**Figure 4:**
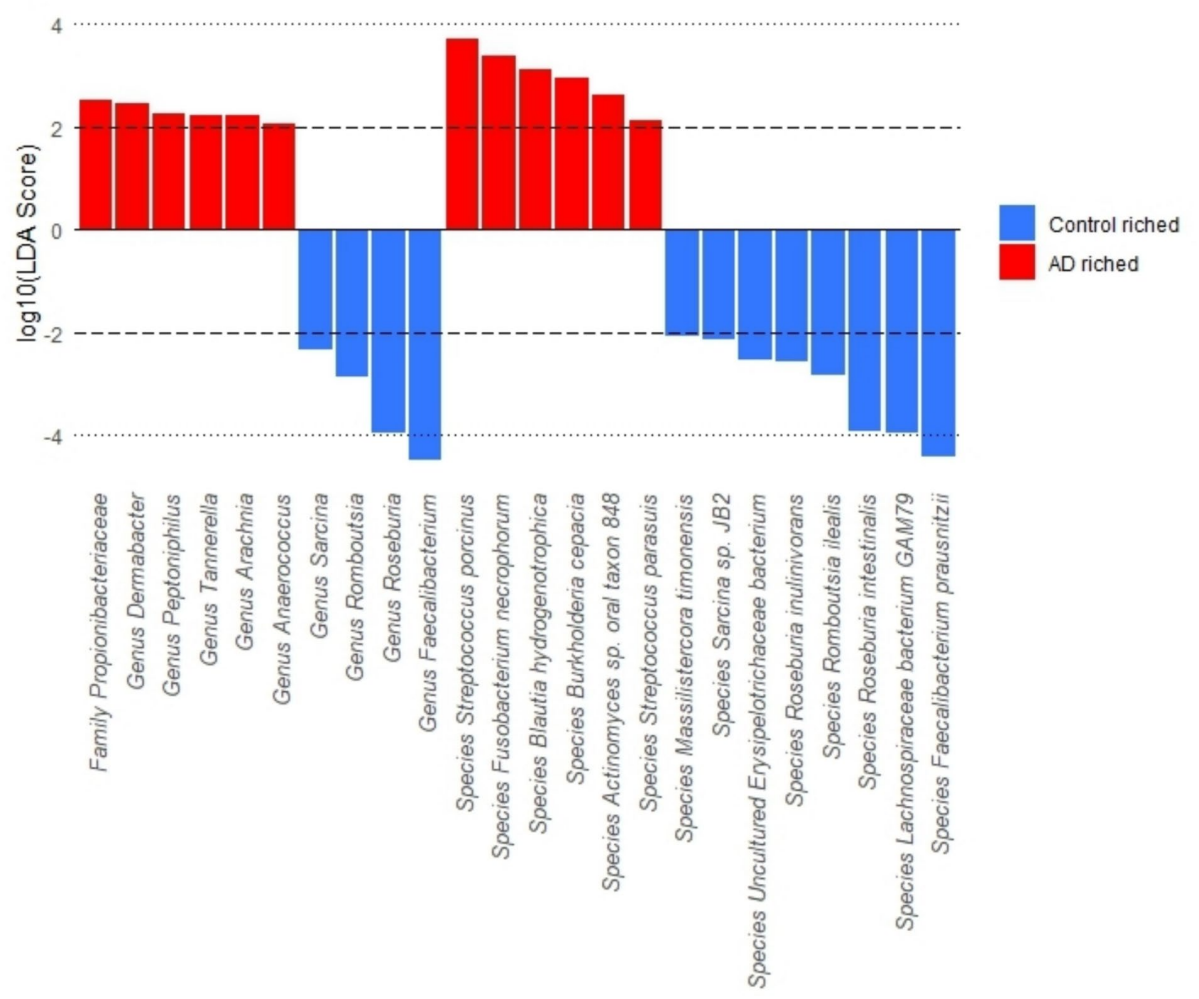
LEfSe analysis of gut microbiota at the family, genus, and species levels.

The results of differences in gut microbiota abundance between the AD group and control group are shown in Figure 4. The findings revealed that the abundance of bacteria belonging to the family *Propionibacteriaceae*, as well as the genera *Peptoniphilus*, Anaerococcus, *Tannerella*, *Arachnia*, and *Dermabacter*, and the species *Burkholderia cepacia*, *Fusobacterium necrophorum*, *Blautia hydrogenotrophica*, *Streptococcus porcinus*, *Streptococcus parasuis*, and *Actinomyces* sp. *oral taxon 848*, were significantly enriched in AD patients. Conversely, the abundance of *Romboutsia*, *Faecalibacterium*, *Roseburia*, *Sarcina* at the genus level, as well as *Massilistercora timonensis*, *Lachnospiraceae bacterium GAM79*, *Romboutsia ilealis*, *Faecalibacterium prausnitzii*, *Roseburia inulinivorans*, *Roseburia intestinalis*, *Sarcina* sp. *JB2*, *Uncultured Erysipelotrichaceae bacterium* at the species level, was significantly enriched in the control group participants.

### 3.5 Correlation between gut microbiota and cognitive scores

The relative abundance of 24 gut microbiota, which were identified as differing between groups, was analyzed using Spearman correlation analysis to assess their correlation with MMSE scores. The findings revealed significant negative correlations between MMSE scores and the relative abundances of the family *Propionibacteriaceae* (*r* = −0.586, *p* < 0.05), genus *Anaerococcus* (*r* = −0.503, *p* < 0.05), genus *Arachnia* (*r* = −0.650, *p* < 0.01), genus *Peptoniphilus* (*r* = −0.710, *p* < 0.01), genus *Tannerella* (*r* = −0.528, *p* < 0.05), species *Actinomyces* sp. *oral taxon 848* (*r* = −0.647, *p* < 0.01), species *Fusobacterium necrophorum* (*r* = −0.607, *p* < 0.05), and species *Streptococcus porcinus* (*r* = −0.444, *p* < 0.05). In contrast, significant positive correlations were observed between MMSE scores and the decreased relative abundances of the genus *Faecalibacterium* (*r* = 0.601, *p* < 0.05), genus *Roseburia* (*r* = 0.562, *p* < 0.05), species *Faecalibacterium prausnitzii* (*r* = 0.601, *p* < 0.05), species *Lachnospiraceae bacterium GAM79* (*r* = 0.529, *p* < 0.05), species *Roseburia intestinalis* (*r* = 0.625, *p* < 0.01), and species *Roseburia inulinivorans* (*r* = 0.579, *p* < 0.05) (Table 3).

**Table 3:** Correlation analysis between gut bacteria abundances and cognitive scores.

Category	Gut bacteria	Correlation coefficient with MMSE score^†^
Family	*Propionibacteriaceae*	−0.586*
Genus	*Anaerococcus*	−0.503*
	*Arachnia*	−0.650**
	*Dermabacter*	−0.454
	*Faecalibacterium*	0.601*
	*Peptoniphilus*	−0.710**
	*Romboutsia*	0.432
	*Roseburia*	0.562*
	*Sarcina*	0.495
	*Tannerella*	−0.528*
Species	*Actinomyces* sp. *oral taxon 848*	−0.647**
	*Blautia hydrogenotrophica*	−0.447
	*Burkholderia cepacia*	−0.375
	*Faecalibacterium prausnitzii*	0.601*
	*Fusobacterium necrophorum*	−0.607*
	*Lachnospiraceae bacterium GAM79*	0.529*
	*Massilistercora timonensis*	0.473
	*Romboutsia ilealis*	0.417
	*Roseburia intestinalis*	0.625**
	*Roseburia inulinivorans*	0.579*
	*Sarcina* sp. *JB2*	0.495
	*Streptococcus parasuis*	−0.444
	*Streptococcus porcinus*	−0.591*
	*Uncultured Erysipelotrichaceae bacterium*	0.457

^†^Spearman correlation analysis; *p-value < 0.05, **p-value < 0.01.

### 3.6 Correlation between miRNAs, gut microbiota, and blood albumin level

Blood albumin level was found to have statistical differences between the AD group and the control group, and the impact of blood albumin level on the results of this study could not be ruled out. Therefore, for the miRNAs with differential expression between groups and the gut bacteria with differential abundances between groups, we also analyzed their correlations with blood albumin level. We found that genus *Faecalibacterium* (*r* = 0.503, *p* < 0.05), species *Faecalibacterium prausnitzii* (*r* = 0.503, *p* < 0.05), and species *Lachnospiraceae bacterium GAM79* (*r* = 0.509, *p* < 0.05) showed significant positive correlations with blood albumin level (Supplementary Table S2). Additionally, hsa-miR-323a-5p (*r* = 0.532, *p* < 0.05) also demonstrated a notable positive correlation with blood albumin level (Supplementary Table S3).

### 3.7 Correlation between plasma exosomal miRNA and gut microbiota

**Figure 5:**
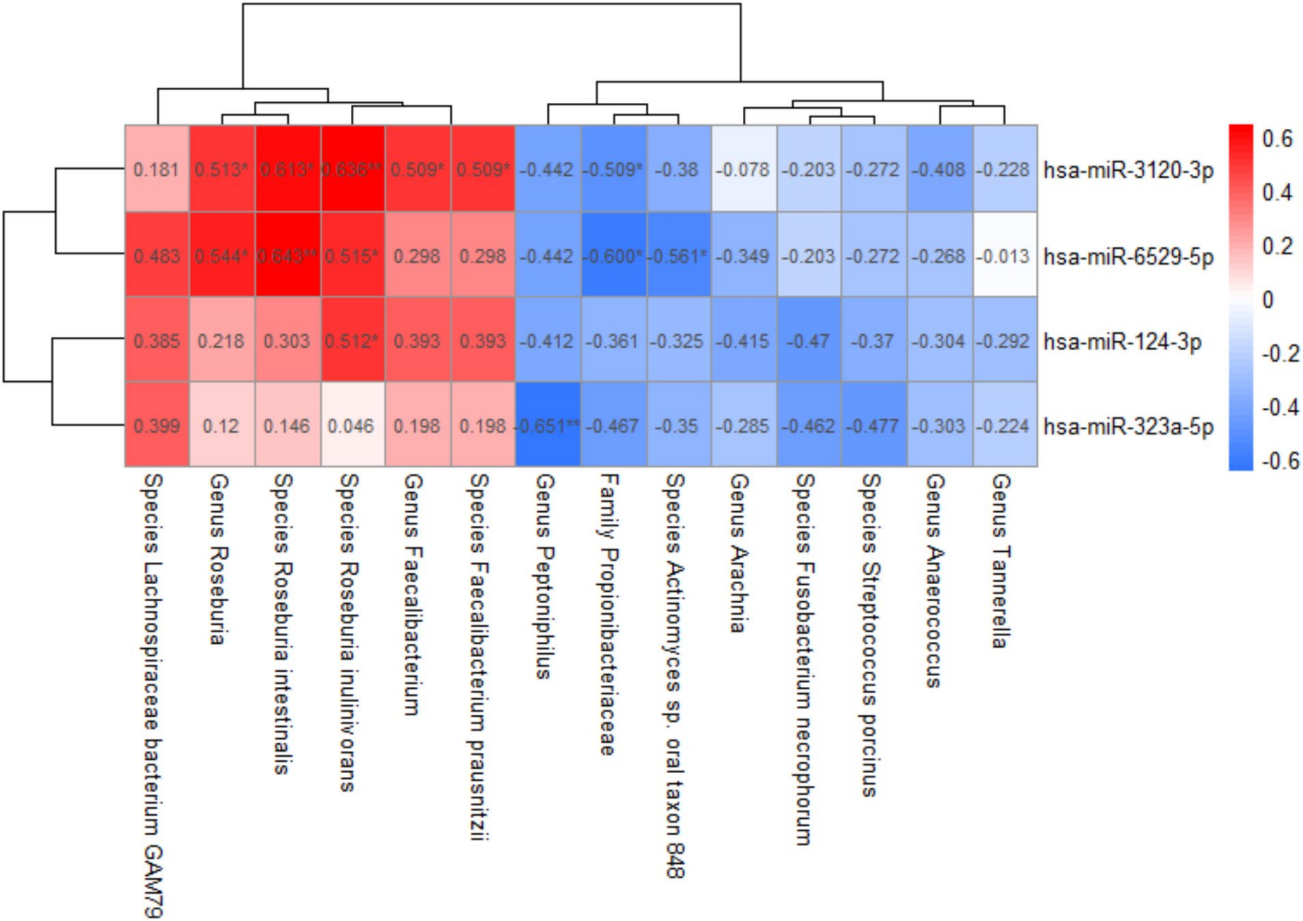
Correlation between plasma exosomal miRNAs and gut microbiota. Spearman correlation analysis was used; *p-value < 0.05, **p-value < 0.01.

From the above, we identified four group-differentially expressed plasma exosomal miRNAs and 14 group-differentially abundant gut microbiota taxa, both significantly correlated with cognitive scores. The correlations between them were further analyzed (Figure 5). The abundance of *Propionibacteriaceae*, *Faecalibacterium*, *Roseburia*, *Faecalibacterium prausnitzii*, *Roseburia intestinalis*, and *Roseburia inulinivorans* was found to be significantly associated with hsa-miR-3120-3p. Additionally, the abundance of *Propionibacteriaceae*, *Roseburia*, *Actinomyces* sp. *oral taxon 848*, *Roseburia intestinalis*, and *Roseburia inulinivorans* was significantly associated with hsa-miR-6529-5p, while *Roseburia inulinivorans* was positively correlated with hsa-miR-124-3p, and *Peptoniphilus* exhibited a negative correlation with hsa-miR-323a-5p.

## 4 Discussion

In this study, we preliminarily explored the correlations between plasma exosomal miRNAs, gut microbiota, and cognitive impairment. In patients with AD, we identified 10 plasma exosomal miRNAs with differential expression and 24 gut microbiota taxa with differential abundances. Among them, the expression levels of 4 exosomal miRNAs and the relative abundances of 14 gut microbiota taxa were found to have significant correlations with MMSE scores. Notably, the abundance of potential probiotics, specifically *Roseburia intestinalis*, *Roseburia inulinivorans*, and *Faecalibacterium prausnitzii*, which was found to be decreased in AD patients, exhibited positive correlations with specific exosomal miRNAs.

The levels of four downregulated plasma exosomal miRNAs in AD patients, including miR-124-3p, −3,120-3p, −6,529-5p, and -323a-5p, were found to be positively correlated with MMSE scores, suggesting that these miRNAs could be involved in the process of cognitive impairment. Some studies indicated that exosomes are associated with neurodevelopment and neuroinflammation (Reza-Zaldivar et al., 2019; Sharma et al., 2019; Zhang T. et al., 2021). A previous study has ascertained that exosomes, enriched with miRNAs that facilitate neurogenesis, such as miR-17-92, display a neurorestorative effect on neural injuries after ischemia (Xin et al., 2017). miR-124-3p, a microRNA highly abundant in brain tissue, targets genes involved in the synthesis of BACE1. A study has shown that it inhibited the upregulation of BACE1 protein and the overexpression of Aβ in a low perfusion brain model (Zhang et al., 2017), indicating a close association with AD pathology. Furthermore, in the rmTBI mouse model, microglial-derived exosomes enriched with miR-124-3p were found to be not only internalized by neurons in the injured brain tissue but also transported to hippocampal neurons, thereby mitigating the neurodegenerative process via the Rela/ApoE signaling pathway (Ge et al., 2020). Additionally, *in vitro* experiments and studies conducted on AD model mice have demonstrated that the absence of miR-124-3p could promote the hyperphosphorylation of tau protein, resulting in neurodegenerative changes (Zhou et al., 2019). Our study found that miR-124-3p levels were downregulated in the plasma exosomes of AD patients, suggesting a diminished neuroprotective function.

miR-3120 is a signaling RNA that targets heat shock protein 70 and auxilin, modulating the uncoating of clathrin-coated vesicles (Scott et al., 2012). It is also a potential biomarker for head and neck epithelial cell carcinomas (Shimada et al., 2021), but there is limited research on its role in neuroscience. KEGG pathway enrichment analysis revealed that target genes of miR-3120-3p were associated with nerve growth factor and bacterial invasion, while GO enrichment analysis demonstrated that its target genes were enriched in functions such as neural system development, protein K48-linked ubiquitination, JNK signaling pathway, and JUN kinase activation. The process of protein K48-linked ubiquitination has been discovered to be associated with the recognition and clearance of soluble misfolded proteins (Mallette and Richard, 2012). The pathogenesis of AD is thought to involve the misfolding of proteins, the formation of soluble protein oligomers, and the phosphorylation of tau protein (Haass and Selkoe, 2007). Furthermore, prior research has demonstrated a correlation between the activation of JNK and Aβ in brain tissue (Shoji et al., 2000), and it has been discovered that tumor necrosis factor-*α*, interleukin, and interferon-*γ* can stimulate the JNK-dependent MAPK pathway, which is involved in the clearance of APP (Liao et al., 2004).

miR-323a-5p has been found to inhibit the proliferation of neuroblastoma cells (Soriano et al., 2019), while lncRNA SNHG7 could sponge miR-323a-5p and promote neuroblastoma progression (Jia et al., 2020). Functional enrichment analysis of the predicted target genes of miR-323a-5p in this study revealed a relationship with the APP cleavage process, which generates AD-associated pathogenic proteins. However, there is still limited research on miR-323a-5p in this area. Additionally, we found that GO analysis of the predicted target genes of hsa-miR-323a-5p, −6,529-5p, and −124-3p were all related to the SNARE complex, whose deficiencies are associated with the proteomic pathological changes in AD (Karmakar et al., 2019). In a word, the functional enrichment analysis of these miRNA target genes implies a close link between these miRNAs and the pathogenesis of AD. However, the specific regulatory mechanisms of their roles still require further research to explore.

Our metagenomic sequencing results revealed a significant decrease in the relative abundance of microbiota producing short-chain fatty acids (SCFAs) in AD patients, particularly the genus *Faecalibacterium* and its species *Faecalibacterium prausnitzii*, as well as the genus *Roseburia* and its species, *Roseburia inulinivorans* and *Roseburia intestinalis*. Similarly, Sheng et al. reported a decreasing trend in the relative abundance of *Faecalibacterium* from normal cognitive function to perceived cognitive decline and subsequently to mild neurocognitive impairment (Sheng et al., 2021). This study also revealed a negative correlation between the abundance of *Faecalibacterium* and brain amyloid-beta load in cognitively normal volunteers (Sheng et al., 2022), suggesting that a decrease in the abundance of *Faecalibacterium* may be a pathogenic factor in cognitive disorders such as AD. Research conducted by Ueda et al. found a reduction in *Faecalibacterium prausnitzii* among participants with mild cognitive impairment and demonstrated that strains of *Faecalibacterium prausnitzii* isolated from healthy volunteers could ameliorate the Aβ-induced cognitive impairments in mice (Ueda et al., 2021).

Research conducted in Kazakhstan also found a decrease in the abundance of *Roseburia* in AD patients (Kaiyrlykyzy et al., 2022). Bacteria of genus Roseburia, and species *Roseburia inulinivorans* and *Roseburia intestinalis*, are capable of producing SCFAs, which can affect immune balance, inflammatory responses, and other physiological processes (Tamanai-Shacoori et al., 2017). SCFAs have been shown to influence neuroinflammation by inhibiting the expression of pro-inflammatory cytokines, while simultaneously promoting microglial maturation and function, which may be beneficial for AD (Liu et al., 2020; Wenzel et al., 2020). *Roseburia intestinalis*, one of the primary butyrate-producing bacteria in the human gut, has been found to inhibit the expression of interleukin-17 (IL-17) in mouse models and *in vitro* cellular studies, thereby reducing inflammation (Zhu et al., 2018). In the central nervous system, IL-17 can induce neuronal damage either alone or in synergy with other factors (Waisman et al., 2015). Our current study observed a reduced abundance of *Roseburia intestinalis* in AD patients, which may be due to reduced inhibition of IL-17 expression, thereby promoting neuroinflammation and neuronal damage. Therefore, SCFAs-producing probiotics, namely *Faecalibacterium* and *Roseburia*, may have a protective effect on AD and represent potential therapeutic targets.

Our research findings also revealed significant enrichment of *Propionibacteriaceae*, *Peptoniphilus*, *Arachnia*, *Tannerella*, as well as species including *Streptococcus porcinus*, *Fusobacterium necrophorum*, and *Actinomyces* sp. *oral taxon 848* in AD patients, all of which showed a significant negative correlation with MMSE scores. *Tannerella*, *Fusobacterium necrophorum*, and *Actinomyces* sp. *oral taxon 848* can inhabit the human oral cavity and are potential oral pathogens. Studies have shown that some oral pathogenic bacteria can invade the brain, where they produce amyloid proteins leading to Aβ deposition, thereby inducing or exacerbating AD (Poole et al., 2013; Singhrao et al., 2014; Sureda et al., 2020). In addition, a community-based study of multiracial elderly individuals found an increased risk of AD among participants with high serum IgG antibodies against *Actinomyces naeslundii* (Noble et al., 2014). In our study, these enriched gut microbiota in AD patients may be involved in the pathogenesis of AD. However, relevant research is currently lacking, and the underlying mechanisms of their roles in AD remain to be further investigated.

We also identified significant associations between gut microbiota and plasma exosomal miRNAs. These findings further support the microbiota-gut-brain axis theory, which proposes that there is a crosstalk between gut microbiota and the brain, jointly influencing the physiological functions of the body. In AD patients, intestinal dysbiosis, intestinal barrier damage, and the increase of pro-inflammatory bacteria lead to more release of toxins or metabolites such as lipopolysaccharide and amyloid into the bloodstream (Ayyanar and Vijayan, 2024). These harmful substances can subsequently cross the BBB and enter neurons, activating signals such as NF-κB and mediating the expression of specific miRNAs, thereby causing neuroinflammation and neurodegeneration (Ayyanar and Vijayan, 2024). Studies have shown that activated microglia and astrocytes secrete exosomes rich in miRNAs, which can enhance neuroinflammation, activate complement system, impair innate immune signaling transduction, and exacerbate disease progression (Lukiw and Pogue, 2020). Therefore, the exosomal miRNAs released from brain tissue into the bloodstream reflect the pathological state of AD. This is also one of the reasons why certain plasma exosomal miRNAs are considered biomarkers for AD. In this study, we found a decrease in the abundance of SCFAs-producing probiotics (such as *Roseburia* and *Faecalibacterium*) in AD patients, which was positively correlated with the decreased expression of exosomal miR-124-3p, miR-3120-3p, or miR-6529-5p. This may indicate a decrease in the levels of SCFAs derived from probiotics in AD patients, which in turn diminishes their inhibitory effect on neuroinflammation. Consequently, this could also result in decreased expression of some miRNAs (such as miR-124-3p) that exert protective effects against AD.

This study initially investigated the potential correlation between plasma exosomal miRNAs and gut microbiota in the development of AD. However, this study still has some shortcomings. First, the latest 2011 NIA-AA diagnostic criteria (McKhann et al., 2011) were not used, as some patients did not cooperate with the examination, so the 1984 NINCDS-ADRDA criteria were used. Second, this study could not determine the organ or tissue source of the plasma exosomes extracted. Thirdly, as a preliminary exploratory study, this research was a cross-sectional study with a relatively small sample size, which limited the possibility of causal inference. The results obtained still need to be validated by cohort studies with large sample sizes. Lastly, a notable disparity in plasma albumin levels was observed between the AD group and the cognitively normal group. A previous study demonstrated that plasma exchange, when coupled with albumin replacement therapy, could decelerate cognitive deterioration in patients with AD (Boada et al., 2020). This suggests that albumin might act as a confounding variable. Nevertheless, most of the significantly altered miRNAs and gut microbiota in this study were not significantly correlated with albumin.

## 5 Conclusion

The study revealed a close association between gut microbiota and plasma exosomal miRNAs in AD patients, suggesting their potential as biomarkers and therapeutic targets for AD. Future studies should further explore the application value of these biomarkers in the early screening, diagnosis, and treatment of AD, as well as their specific mechanisms of action in the pathological processes of AD.

## Data Availability

The raw data from plasma exosomal miRNA sequencing and fecal metagenomic sequencing have been deposited in the National Center for Biotechnology Information’s (NCBI) Sequence Read Archive (SRA) under the BioProject accession numbers PRJNA1221222 (URL: https://www.ncbi.nlm.nih.gov/bioproject/PRJNA1221222) and PRJNA1221292 (URL: https://www.ncbi.nlm.nih.gov/bioproject/PRJNA1221292), respectively. Additionally, the individual sample accession numbers for these two BioProjects range from SRR32277269 to SRR32277284 and from SRR32280283 to SRR32280298. These samples’ raw data can be accessed through the SRA website at https://www.ncbi.nlm.nih.gov/sra. For instance, SRR32277269 can be found at: https://www.ncbi.nlm.nih.gov/sra/SRR32277269.

## Footnotes

^1^ https://www.mirbase.org/

^2^ http://mirwalk.umm.uni-heidelberg.de/

^3^ http://huttenhower.sph.harvard.edu/galaxy/
