# Supplementary material for: Plasma exosomal miRNA expression and gut microbiota dysbiosis are associated with cognitive impairment in Alzheimer’s disease
Source: Front Neurosci. 2025 Feb 19;19:1545690. doi: 10.3389/fnins.2025.1545690 (PMC11880238; doi:10.3389/fnins.2025.1545690)
Supplement: Supplementary file 1 [file Table_1.docx]

**Table S1.** Functional enrichment analysis of target genes predicted by key plasma exosomal microRNAs

| microRNA | The number of predicted target genes | Functional enrichment analysis | Enriched pathways and functions (adjusted p value＜0.05) |
| --- | --- | --- | --- |
| hsa-miR-3120-3p | 2097 | KEGG | hsa05100 Bacterial invasion of epithelial cells |
|  |  |  | hsa04722 Neurotrophin signaling pathway |
|  |  | GO: MF | GO:0003924 GTPase activity |
|  |  |  | GO:0004672 protein kinase activity |
|  |  |  | GO:0004674 protein serinethreonine kinase activity |
|  |  |  | GO:0005516 calmodulin binding |
|  |  |  | GO:0002151 G-quadruplex RNA binding |
|  |  |  | GO:0005249 voltage-gated potassium channel activity |
|  |  |  | GO:0042813 Wnt-activated receptor activity |
|  |  |  | GO:0004709 MAP kinase kinase kinase activity |
|  |  |  | GO:0001228 DNA-binding transcription activator activity，RNA polymerase II-specific |
|  |  |  | GO:0017147 Wnt-protein binding |
|  |  |  | GO:0003700 DNA-binding transcription factor activity |
|  |  |  | GO:0045296 cadherin binding |
|  |  |  | GO:0003729 mRNA binding |
|  |  |  | GO:0106311 protein threonine kinase activity |
|  |  |  | GO:0004683 calmodulin-dependent protein kinase activity |
|  |  |  | GO:0106310 protein serine kinase activity |
|  |  |  | GO:0031624 ubiquitin conjugating enzyme binding |
|  |  |  | GO:0004708 MAP kinase kinase activity |
|  |  |  | GO:0000987 cis-regulatory region sequence-specific DNA binding |
|  |  |  | GO:0017124 SH3 domain binding |
|  |  |  | GO:0000014 single-stranded DNA endodeoxyribonuclease activity |
|  |  |  | GO:0005432 calcium:sodium antiporter activity |
|  |  | GO: CC | GO:0005925 focal adhesion |
|  |  |  | GO:0000139 Golgi membrane |
|  |  |  | GO:0005768 endosome |
|  |  |  | GO:0005770 late endosome |
|  |  |  | GO:0030424 axon |
|  |  |  | GO:0030870 Mre11 complex |
|  |  |  | GO:0031901 early endosome membrane |
|  |  |  | GO:0032991 protein-containing complex |
|  |  |  | GO:0044224 juxtaparanode region of axon |
|  |  |  | GO:0001518 voltage-gated sodium channel complex |
|  |  |  | GO:0014069 postsynaptic density |
|  |  |  | GO:0098978 glutamatergic synapse |
|  |  |  | GO:0043005 neuron projection |
|  |  |  | GO:0055037 recycling endosome |
|  |  | GO: BP | GO:0006468 protein phosphorylation |
|  |  |  | GO:0046777 protein autophosphorylation |
|  |  |  | GO:0006355 regulation of transcription, DNA-templated |
|  |  |  | GO:0007254 JNK cascade |
|  |  |  | GO:0007399 nervous system development |
|  |  |  | GO:0070936 protein K48-linked ubiquitination |
|  |  |  | GO:0061003 positive regulation of dendritic spine morphogenesis |
|  |  |  | GO:0007173 epidermal growth factor receptor signaling pathway |
|  |  |  | GO:0045893 positive regulation of transcription, DNA-templated |
|  |  |  | GO:0007257 activation of JUN kinase activity |
|  |  |  | GO:0008053 mitochondrial fusion |
| hsa-miR-6529-5p | 3981 | GO: MF | GO:0044325 ion channel binding |
|  |  |  | GO:0003729 mRNA binding |
|  |  |  | GO:0061630 ubiquitin protein ligase activity |
|  |  | GO: CC | GO:0008021 synaptic vesicle |
|  |  |  | GO:0000139 Golgi membrane |
|  |  |  | GO:0031201 SNARE complex |
|  |  |  | GO:0043005 neuron projection |
|  |  |  | GO:0043197 dendritic spine |
|  |  |  | GO:0005802 trans-Golgi network |
|  |  |  | GO:0030424 axon |
|  |  | GO: BP | GO:0007399 nervous system development |
|  |  |  | GO:0051092 positive regulation of NF-kappaB transcription factor activity |
|  |  |  | GO:0000209 protein polyubiquitination |
|  |  |  | GO:0008284 positive regulation of cell population proliferation |
|  |  |  | GO:0016239 positive regulation of macroautophagy |
|  |  |  | GO:0043123 positive regulation of I-kappaB kinaseNF-kappaB signaling |
|  |  |  | GO:0048013 ephrin receptor signaling pathway |
|  |  |  | GO:0060271 cilium assembly |
|  |  |  | GO:0061024 membrane organization |
| hsa-miR-124-3p | 2890 | GO: MF | GO:0004674 protein serinethreonine kinase activity |
|  |  |  | GO:0003713 transcription coactivator activity |
|  |  |  | GO:0004708 MAP kinase kinase activity |
|  |  |  | GO:0005516 calmodulin binding |
|  |  |  | GO:0051015 actin filament binding |
|  |  |  | GO:0000149 SNARE binding |
|  |  |  | GO:0004115 3',5'-cyclic-AMP phosphodiesterase activity |
|  |  |  | GO:0004842 ubiquitin-protein transferase activity |
|  |  |  | GO:0005096 GTPase activator activity |
|  |  |  | GO:0019901 protein kinase binding |
|  |  |  | GO:0061630 ubiquitin protein ligase activity |
|  |  | GO: BP | GO:0000187 activation of MAPK activity |
|  |  |  | GO:0032456 endocytic recycling |
|  |  |  | GO:0006468 protein phosphorylation |
|  |  |  | GO:0045893 positive regulation of transcription, DNA-templated |
|  |  |  | GO:0006511 ubiquitin-dependent protein catabolic process |
|  |  |  | GO:0072659 protein localization to plasma membrane |
|  |  |  | GO:0006486 protein glycosylation |
|  |  |  | GO:0016567 protein ubiquitination |
| hsa-miR-323a-5p | 1855 | GO: CC | GO:0000139 Golgi membrane |
|  |  |  | GO:0005741 mitochondrial outer membrane |
|  |  |  | GO:0016592 mediator complex |
|  |  |  | GO:0031201 SNARE complex |
|  |  | GO: BP | GO:0097194 execution phase of apoptosis |
|  |  |  | GO:0019852 L-ascorbic acid metabolic process |
|  |  |  | GO:0042987 amyloid precursor protein catabolic process |

Note: KEGG: Kyoto Encyclopedia of Genes and Genomes; GO: Gene Ontology; BP: Biological Process; MF: Molecular Function; CC: Cellular Component.
