# Supplementary material for: Plasma exosomal miRNA expression and gut microbiota dysbiosis are associated with cognitive impairment in Alzheimer’s disease
Source: Front Neurosci. 2025 Feb 19;19:1545690. doi: 10.3389/fnins.2025.1545690 (PMC11880238; doi:10.3389/fnins.2025.1545690)
Supplement: Supplementary file 2 [file Table_2.docx]

**Table S2.** Correlation analysis between gut microbiota abundances and blood albumin level

| Category | Gut microbiota | Correlation coefficient **^†^** |
| --- | --- | --- |
| Family | Propionibacteriaceae | -0.418 |
| Genus | Anaerococcus | -0.307 |
|  | Arachnia | -0.401^P^ |
|  | Dermabacter | -0.35 |
|  | Faecalibacterium | 0.503* |
|  | Peptoniphilus | -0.41 |
|  | Romboutsia | 0.318 |
|  | Roseburia | 0.379 |
|  | Sarcina | 0.436 |
|  | Tannerella | -0.119 |
| Species | Actinomyces sp. oral taxon 848 | -0.445^P^ |
|  | Blautia hydrogenotrophica | 0.062 |
|  | Burkholderia cepacia | -0.058 |
|  | Faecalibacterium prausnitzii | 0.503* |
|  | Fusobacterium necrophorum | -0.346 |
|  | Lachnospiraceae bacterium GAM79 | 0.509* |
|  | Massilistercora timonensis | 0.355 |
|  | Romboutsia ilealis | 0.294 |
|  | Roseburia intestinalis | 0.444 |
|  | Roseburia inulinivorans | 0.307 |
|  | Sarcina sp. JB2 | 0.436 |
|  | Streptococcus parasuis | -0.264 |
|  | Streptococcus porcinus | -0.263 |
|  | Uncultured Erysipelotrichaceae bacterium | 0.495 |

Note：**^†^** For gut microbiota with a normal distribution of relative abundance, Pearson correlation analysis was used; otherwise, Spearman correlation analysis was employed. ^P^ Pearson correlation analysis; * p value<0.05.
