# Supplementary material for: Plasma exosomal miRNA expression and gut microbiota dysbiosis are associated with cognitive impairment in Alzheimer’s disease
Source: Front Neurosci. 2025 Feb 19;19:1545690. doi: 10.3389/fnins.2025.1545690 (PMC11880238; doi:10.3389/fnins.2025.1545690)
Supplement: Supplementary file 3 [file Table_3.docx]

**Table S3.** Correlation analysis between plasma exosomal microRNA expressions and blood albumin level

| microRNAs | Correlation coefficient**^†^** |
| --- | --- |
| hsa-miR-124-3p | 0.264 |
| hsa-miR-7-2-3p | -0.196 |
| hsa-miR-6826-3p | -0.248 |
| hsa-miR-6769b-5p | -0.196 |
| hsa-miR-676-3p | 0.135 |
| hsa-miR-6731-5p | 0.135 |
| hsa-miR-6529-5p | 0.078 |
| hsa-miR-627-5p | 0.084 |
| hsa-miR-323a-5p | 0.532* |
| hsa-miR-3120-3p | 0.108 |

Note: **^†^** Spearman correlation analysis; * p value<0.05.
