# Supplementary figures and images for: Plasma exosomal miRNA expression and gut microbiota dysbiosis are associated with cognitive impairment in Alzheimer’s disease
Source: Front Neurosci. 2025 Feb 19;19:1545690. doi: 10.3389/fnins.2025.1545690 (PMC11880238; doi:10.3389/fnins.2025.1545690)

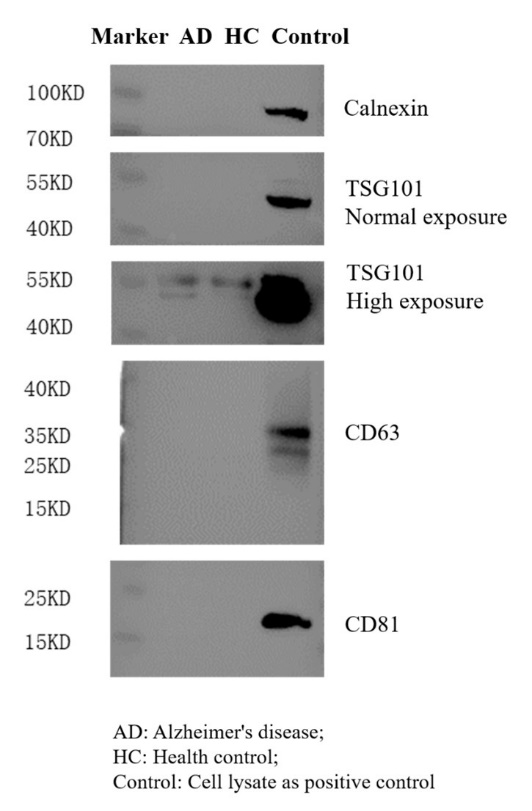


**Figure S1.** Detecting plasma exosome marker proteins using Western blot

Supplement: Supplementary file 4 [file Table_4.docx]
